# Adaptation-Dependent Synchronous Activity Contributes to Receptive Field Size Change of Bullfrog Retinal Ganglion Cell

**DOI:** 10.1371/journal.pone.0034336

**Published:** 2012-03-27

**Authors:** Hao Li, Wen-Zhong Liu, Pei-Ji Liang

**Affiliations:** Department of Biomedical Engineering, Shanghai Jiao Tong University, Shanghai, China; Dalhousie University, Canada

## Abstract

Nearby retinal ganglion cells of similar functional subtype have a tendency to discharge spikes in synchrony. The synchronized activity is involved in encoding some aspects of visual input. On the other hand, neurons always continuously adjust their activities in adaptation to some features of visual stimulation, including mean ambient light, contrast level, etc. Previous studies on adaptation were primarily focused on single neuronal activity, however, it is also intriguing to investigate the adaptation process in population neuronal activities. In the present study, by using multi-electrode recording system, we simultaneously recorded spike discharges from a group of dimming detectors (OFF-sustained type ganglion cells) in bullfrog retina. The changes in receptive field properties and synchronization strength during contrast adaptation were analyzed. It was found that, when perfused using normal Ringer's solution, single neuronal receptive field size was reduced during contrast adaptation, which was accompanied by weakening in synchronization strength between adjacent neurons' activities. When dopamine (1 µM) was applied, the adaptation-related receptive field area shrinkage and synchronization weakening were both eliminated. The activation of D1 receptor was involved in the adaptation-related modulation of synchronization and receptive field. Our results thus suggest that the size of single neuron's receptive field is positively related to the strength of its synchronized activity with its neighboring neurons, and the dopaminergic pathway is responsible for the modulation of receptive field property and synchronous activity of the ganglion cells during the adaptation process.

## Introduction

Visual system operates under a wide range of light conditions with its limited range of firing response. In this sense, adaptation can benefit the system encoding visual information under various visual environments for saving energy and improving signaling capability [1], [2]. At the earliest stages of visual system, i.e., in the retina and the lateral geniculate nucleus (LGN), neurons adapt to some properties of input light including the mean light intensity and contrast [3], [4], [5], [6]. In retinal ganglion cells (RGCs), adaptation to contrast has been well observed when the retina is exposed to sustained high contrast stimulus, in a sense that the onset of stimulus elicits high rate firing from the cell, and the firing rate is decreased progressively to a steady level which is much lower than its transient response [4], [7]. Up to date, investigations on adaptation have mainly been concentrated on single cell activity, including adaptation occurrence with different time scale [4]; however, little attention has been paid to population activities changes during the adaptation process.

Adjacent retinal ganglion cells (RGCs) are often engaged in concerted spiking activities, which can be categorized into several subtypes based on neuronal wiring [8], [9]. Correlated activity between retinal ganglion cells induced by common inputs from presynaptic bipolar and amacrine cells is characterized by distributed time lags in the cross-correlogram between pair-wise neuronal firing sequences; while the precisely synchronized activity between the neighboring ganglion cells mediated by gap junctions is characterized by a sharp peak at zero-lag in the cross-correlogram, in which case ganglion cells fire synchronously with a temporal precision of a couple of milliseconds.

In addition to its contribution in the excitatory signal sharing among adjacent neurons, electrical coupling among retinal neurons also contributes to the extension of their receptive fields (RF). Evidence was found in horizontal cells [10], [11], amacrine cells [12] and bipolar cells [13]. It is thus reasonable to make an inference that the receptive field of RGC is also partly dependent on the gap junctions among adjacent RGCs [14]. Interestingly, it was also reported that receptive field of ganglion cell can change due to light adaptation status [15], [16], [17].

In the present study, using multi-electrode recording technique, we examined the changes of synchronous activity between dimming detectors (OFF-sustained type RGC) and receptive field size changes of these neurons during contrast adaptation in bullfrog retina. It was found that, for most dimming detectors recorded in our experiments, the cell's receptive field size was reduced during the adaptation process, which was accompanied by a decreased strength of synchronized activity. Further investigation with application of dopamine (DA), a neuromodulator which modulates the gap junctional conductance between ganglion cells [18], and dopamine receptor antagonists suggested that adaptation-related modulations in receptive field size and synchronous activities were regulated through the activation of dopaminergic pathway.

## Materials and Methods

### Preparation

Bullfrogs were dark adapted for 30 minutes prior to the experiments. Isolated retinas were used for electrophysiological experiments in accordance with guidelines for the care and use of animals as prescribed by the Association for Research in Vision and Ophthalmology. Under a dim red light, frog was double pithed and eyes were enucleated. The eyeball was hemisected, and the cornea and lens were separated from the posterior part. The eyecup was cut into several pieces and the retina was isolated carefully from the pigment epithelium [17], [19]. The isolated retina was immediately transferred onto a piece of multi-electrode array (MEA, MCS GmbH, Germany) with the ganglion cell layer contacting the electrodes. The preparation was superfused with normal oxygenated (95% O_2_ and 5% CO_2_) Ringer's solution (containing in mM: 100.0 NaCl, 2.5 KCl, 1.6 MgCl_2_, 2.0 CaCl_2_, 25.0 NaHCO_3_, 10.0 glucose). In pharmacological experiments, dopamine (1 µM), sulpiride (10 µM) and SCH-23390 (10 µM) were applied with the Ringer's solution as desired. All drugs were purchased from Sigma-Aldrich (St. Louis, MO, USA).

### Electrophysiological recording

The multi-electrode array (MEA) consisted of 60 electrodes (10 µm in diameter) arranged in an 8×8 matrix (leaving the 4 corners void). The horizontal and vertical tip-to-tip distances between adjacent electrodes were 100 µm, and the diagonal tip-to-tip distance was 141 µm. The tissue and perfusate were kept under room temperature (22°C–24°C). A small Ag/AgCl pellet with wire was immerged into the bath solution and acted as a reference electrode.

The neuronal photo-responses were recorded simultaneously by the MEA system, and the signals were amplified through a 60-channel amplifier (single-ended, 1,200×, input impedance >10^10^ Ω, output impedance 330 Ω). Signals from the selected channels along with the stimulus were sampled at a rate of 20 kHz (MC Rack, MCS GmbH, Germany) and stored in a computer. Spikes from individual neurons were sorted using principal component analysis [20], [21]. K-means clustering method was then applied to identify the data corresponding to spikes as well as OfflineSorter (Plexon Inc. Texas, USA). In order to get accurate data for spike train analysis, only single-neuron events clarified by all the above mentioned spike-sorting methods were used for further analyses in the present study [22], [23].

### Stimulus and estimation of receptive field properties

Visual stimuli were programmed using MATLAB Psychophysics Toolbox [24] and were displayed on a monitor (796 FD II, MAG, 1024×768 pixels). The visual image was focused to an area of 0.9×0.9 mm^2^ when projected onto the isolated retina via a lens system.

In our experiments, pseudo-random checkerboard flickering sequence was applied, with frame refresh rate of 20 Hz and duration of 250 s. Every frame consisted of 16×16 (row×column) sub-squares (56 µm×56 µm in size), each of which was assigned a value either “1” (12.18 nw/cm^2^) or “−1” (0.00 nw/cm^2^) following an m-sequence. The same checkerboard flickering sequence was applied both in control experiment and during drugs application.

**Figure 1:**
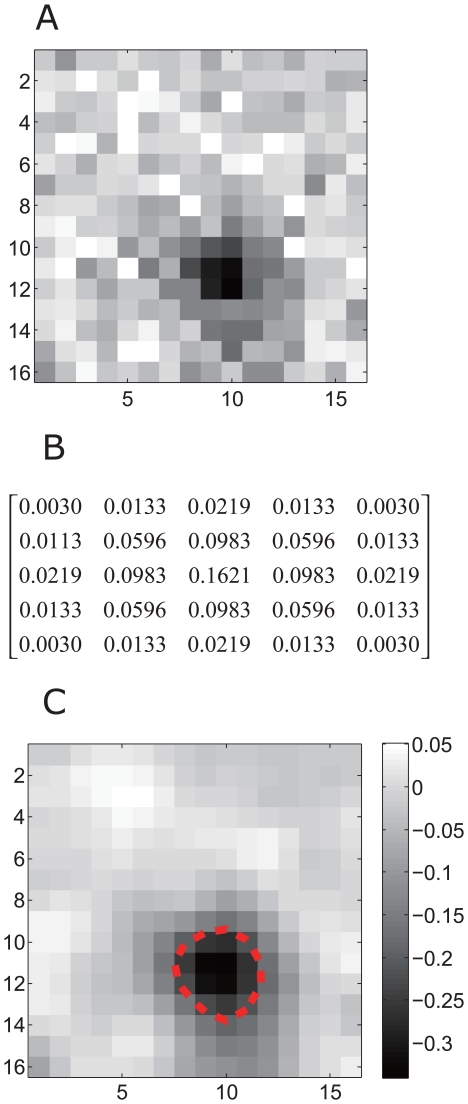
Receptive field of a dimming detector. A: The raw STA of receptive field map. B: The corresponding 5×5 matrix for the Gaussian filter with standard deviation of 1. C: The receptive field was smoothed using a 2-dimensional Gaussian filter, which removed high frequency noise in the original data. The dash-line indicates the estimated boundary of the receptive field.

The size of the RGCs' receptive fields was estimated by calculating the spike-triggered average (STA) according to the neuronal responses to the checker-board stimulation [25]. The original STA image (Fig. 1A) was convoluted with a two-dimensional spatial Gaussian filter (5×5) with the exact values given in Fig. 1B. The filtered receptive field map of an example dimming detector, consisting of 16×16 sub-squares, is plotted in Fig. 1. For the receptive field (RF) border estimation, the 16×16 pixels are converted into a curved surface with two-dimensiaonal interpolation, and the receptive field boundary was determined by the contour line (60%×the minimum negative value) of the curved surface. The area enclosed by the boundary was defined as the receptive field size and quantified in arbitrary units (a.u.) (Fig. 1C, dash-line). It should be emphasized that the receptive field sizes in this study referred exclusively to the receptive field center and not the surround.

### Estimation of the synchronization strength

For a single neuron *a*, cross-correlograms paired with its adjacent cells (recorded by neighboring electrodes with inter-electrode distance ≤400 µm) were computed and normalized as follows:
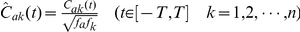
where 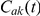 and 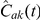 denote the raw and normalized cross-correlogram between neurons *a* and *k*; *t* and *T* denote the time lag and maximal time lag involved in cross-correlogram computation; *f_a_* and *f_k_* are the firing rate of neurons *a* and *k*, respectively; *n* is the number of neurons adjacent to neuron *a*. In our calculation, the strength of synchronization between the neuron pairs was normalized against the firing rates, which eliminated the influence of firing rate on synchronization estimation.

**Figure 2:**
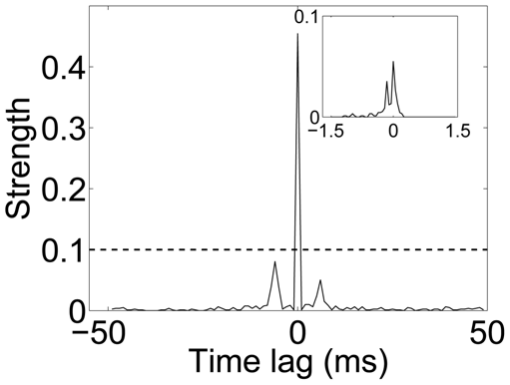
Normalized cross-correlogram of two synchronized ganglion cells. The normalized cross-correlogram, 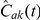, was computed between two neurons with *T* = 50 ms, jitter = 1 ms; inset, *T* = 1.5 ms, jitter = 0.05 ms.

Based on normalized cross-correlogram with jitter (time-bin) of 1 ms, the synchronized pairs were identified as those pairs with the peak value in the normalized cross-correlogram exceeding 0.1 and the width of the central peak less than 2 ms (Fig. 2). The synchronization index was calculated for a neuron if, among its adjacent neurons, there were more than 5 neurons' activities synchronized with it. The synchronization index of neuron *a* (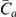) was defined as the mean strength over all synchronized pairs that neuron *a* takes part in:
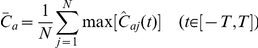
where *N* is the number of the identified synchronized pairs in neuron *a*'s neighboring area with an inter-electrode radius of 400 µm.

## Results

### Dimming detector

Frog retinal ganglion cells can be classified into four subtypes based on their photo-response properties: the dimming detector, the moving-edge detector, the contrast detector, and the convexity detector [26]. In our experiments, only identified dimming detectors (with sustained firings in response to light-off stimulation and with an off-center in receptive field [27]) were analyzed.

### Adaptive change of receptive field size

**Figure 3:**
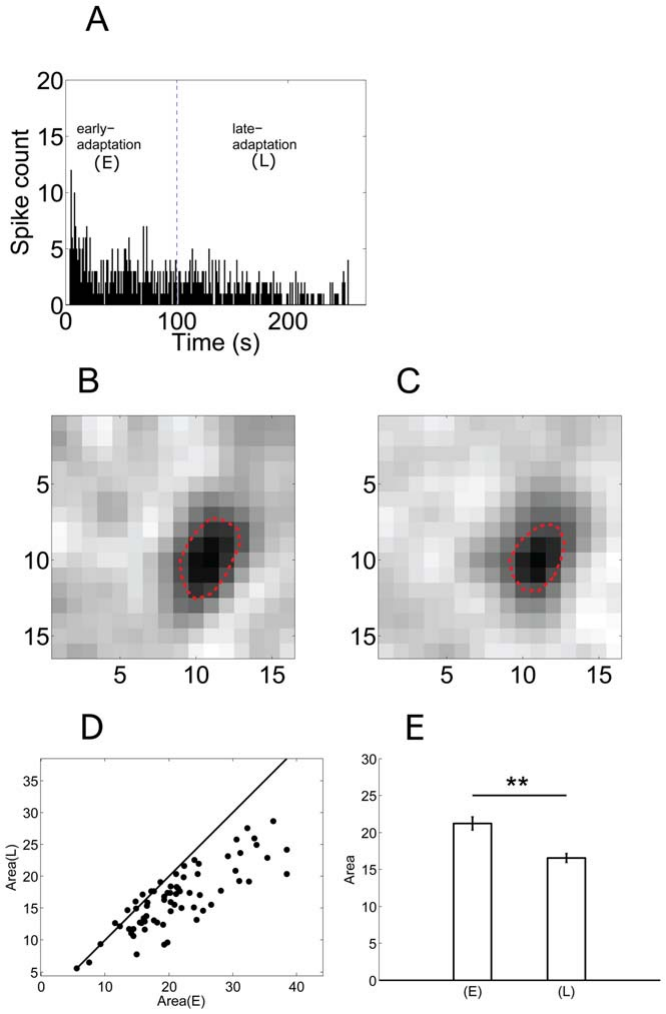
Adaptation-dependent firing rate and receptive field. A: Time-dependent firing rate decline of a dimming detector during contrast-adaptation (bin = 1 s). The dash-line indicates the separation of early- and late-adaptation periods. (E) = early-adaptation; (L) = late-adaptation; B&C: Receptive field area of the same cell in (A) was estimated during early- and late-adaptation, respectively; D: Area(E) vs Area(L) for 70 neurons; E: The average values of the receptive field area. Area(E) = 21.23±0.87 (a.u.), Area(L) = 16.53±0.59 (a.u.) (mean ± S.E., **, p<0.01, paired t-test).

The firing activity of a dimming detector in exposure to sustained pseudo-random checker-board flickering is plotted in Fig. 3A, which is similar to the previously reported contrast-adaptation in retinal ganglion cells [4], [7], [28], [29], [30].

To quantitatively analyze the adaptive change of the neuron's receptive field size, receptive field area was estimated using data recorded during two periods: (1) early-adaptation (0–100 s after the stimulus onset) and (2) late-adaptation (100–250 s after the stimulus onset) (see Fig. 3A, separated by the dash-line). The firing rate during early-adaptation exhibited a gradually descending trend, while the firing rate in late-adaptation basically remained unchanged without obvious increasing/decreasing tendency.

Fig. 3B–C show the receptive field area of the example dimming detector (the same as plotted in Fig. 3A) estimated using data recorded during early-adaptation (Area(E)) and late-adaptation (Area(L)) respectively. The dark region indicated by the closed dash-line shows the receptive field center of this cell, which confirms that this cell is an OFF-center ganglion cell. The cell's receptive field does not show a distinguishable antagonistic surround, which is mainly because that the stimulation we used was not suitable to elicit surround responses [31].

The results plotted in Fig. 3B–C show that the estimated receptive field area was smaller during late-adaptation as compared to that during early-adaptation. In 70 dimming detectors recorded from 10 retinas, receptive field shrinkage was found in 61 (87%) cells (Fig. 3D), and the mean values of the receptive field area decreased significantly (Area(E) = 21.23±0.87 (a.u.), Area(L) = 16.53±0.59 (a.u.) (mean ± S.E.), p<0.01, paired t-test) (Fig. 3E). As receptive field area is closely related to spatial summation of excitatory inputs that a neuron receives [32], the reduced receptive field area suggests that, for the majority of dimming detectors recorded in the present study, the lateral communication among neurons was attenuated during adaptation.

### Synchronized activity

It has been well stated that gap junctional connection is responsible for the synchronized activity between adjacent ganglion cells [9], and the strength of synchronization reflects the strength of gap junctional coupling. In the present study, (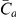 see Methods) is used as an index to describe the strength of gap junctional connection among dimming detectors.

**Figure 4:**
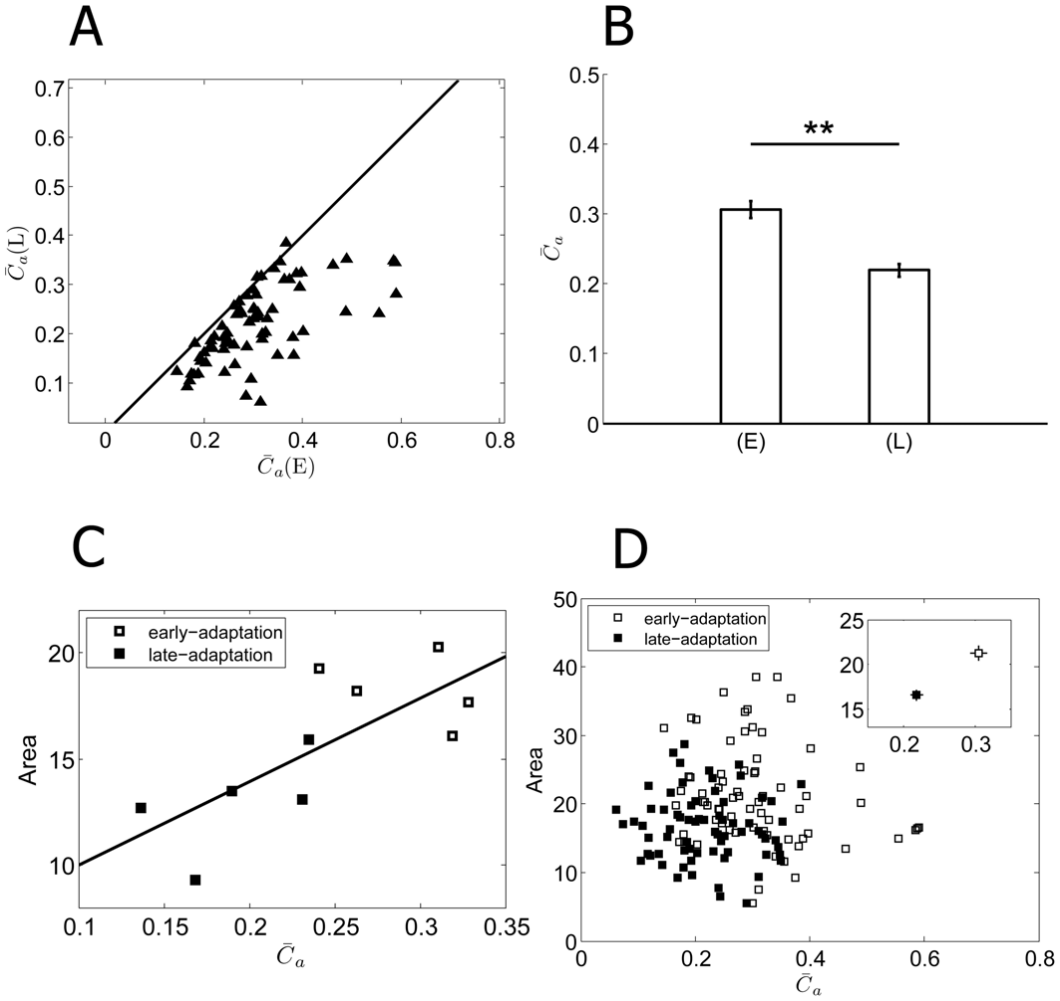
Adaptation-dependent synchronization. A: 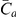 for 70 neurons in early-adaptation (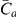
(E)) and late-adaptation (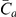
(L)). (E) = early-adaptation; (L) = late-adaptation. B: The averages 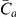
(E) and 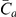
(L) values for the 70 neurons (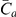
(E) = 0.31±0.01, 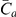
(L) = 0.22±0.01, mean ± S.E., n = 70, **, p<0.01, paired t-tests). C: RF area vs 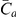 of a retina. Open squares: early-adaptation; filled squares: late-adaptation. The fitted line demonstrates a linear correlation between 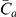 and RF area values. D: RF area vs 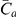 of all the 70 RGCs from 10 retinas.

To investigate the adaptation-dependent changes of synchronization strength, 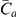 values were calculated using data recorded during early-adaptation (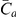
(E)) and late-adaptation (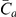
(L)), respectively. Among the 70 neurons recorded from 10 retinas, reduced synchronization index was found in 68 cells (97%, Fig. 4A). The mean values for 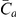
(E) and 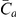
(L) (across the 80 cells) were 0.31±0.01 and 0.22±0.01 (mean ± S.E.) respectively, which were significantly different (p<0.01, paired t-test) (Fig. 4B). Reduced 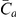 values reflect that the strength of gap junctional connection was decreased during contrast adaptation.

Accompanied by the descending firing rate, the 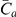 value which describes the population activities also exhibited adaptation-dependent decreasing. Considering the potential relationship between the receptive field size of a neuron and its firing activity in synchrony with its neighbors, we plotted RF area against 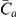. Fig. 4(C) shows the result from a representative retina. The linear association between RF area and 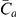 is demonstrated by the fitted line, which indicates the possibility that the lateral gap junctional connections among dimming detectors underlie the extension of receptive field. We noticed that the slope of the fitted line varied among different retinas, therefore a combination of data from 70 cells in 10 retinas exhibited a scattered distribution as shown in Fig. 4(D), while the mean values of RF and 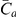 shows the positive correlation (Fig. 4(D), inset).

### Effects of dopaminergic pathway on synchronous activities

Dopamine (DA), as a retinal neural modulator whose release is light-dependent [33], [34], has been implicated in playing key roles in the regulation of gap junctional conductance [18], [35], [36], [37]. In our experiment, exogenous DA (1 µM) was applied to investigate whether the adaption-related Area's shrinkage and 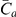 decrease are related to the gap junctional coupling modulated by DA.

**Figure 5:**
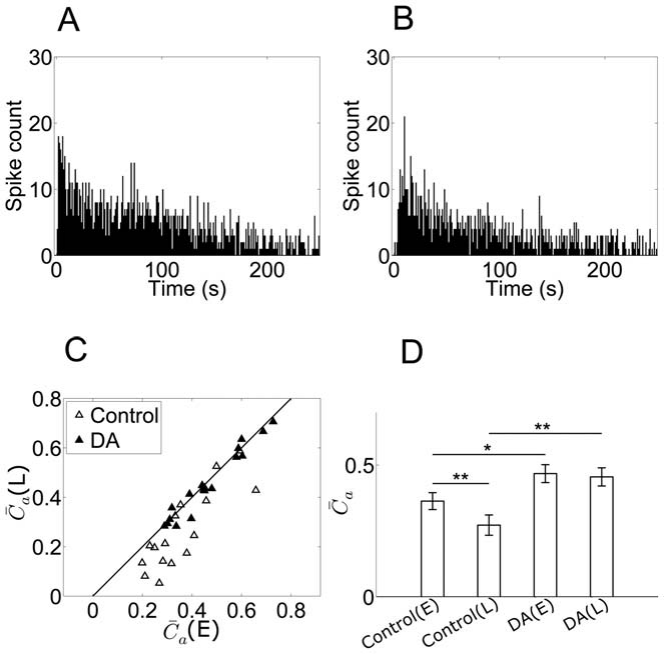
Firing rate and synchronization when DA applied. A&B: Time-dependent firing rate of an example dimming detector during control and DA application, respectively (bin = 1 s); C: The comparison of synchronization strength calculated based on data recorded during early- and late-adaptation during control (open triangles) and DA application (filled triangles). (E) = early-adaptation; (L) = late-adaptation; D: bar plots show mean 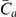 and error bars represent S.E.. Significant differences (based on paired t-tests) are marked by asterisks (*, p<0.02; **, p<0.01).

Fig. 5A–B show the time-dependent firing rate (bin = 1 s) changes of a dimming detector in response to sustained checkerboard flickering, in the normal Ringer's solution and with DA application, respectively. Similar to the control, an obvious firing rate decrease was also observed during DA application, suggesting that the DA application did not change the adaptation process of single cell's firing activity much.

To analyze the possible adaptation-related changes in synchronization strength and receptive field size, the neuronal responses during DA application were segmented into early- and late-adaptation periods as described in Fig. 3A. Relevant 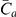 and Area values were calculated. Fig. 5 shows the results for 17 neurons recorded from 6 retinas. During control, decreased 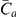 values were found in late-adaptation in 15 neurons (Fig. 5C, open triangle), with the mean 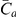
(E) and 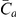
(L) across the sample of 17 cells significantly different (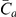
(E) = 0.36±0.03, 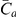
(L) = 0.27±0.04, p<0.01, paired t-test) (Fig. 5D). However, with application of DA, 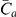 value of the 17 neurons in early- and late-adaptation did not show obvious changes (Fig. 5C, filled triangle) and the mean 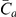
(E) and 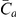
(L) exhibited no significant difference (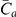
(E) = 0.47±0.03, 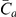
(L) = 0.46±0.03, Fig. 5D). Moreover, the synchronization strength index in the presence of DA was significantly larger for both in early- and late-adaptation periods as compared to control (Fig. 5D). These results suggest that application of DA resulted in a significant strengthening of the gap junctional connections among dimming detectors, and eliminated the adaptation-dependent decrement in 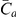.

**Figure 6 pone-0034336-g006:**
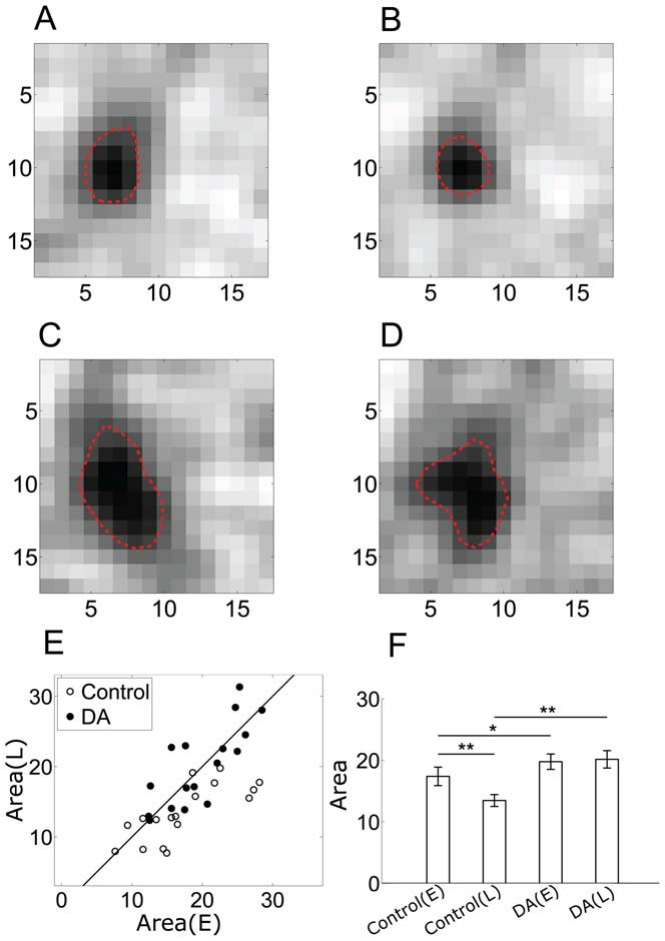
Receptive field under DA condition. (A–D) The receptive area of a dimming detector in early- and late-adaptation under control condition (A&B) and DA condition (C&D). E; Area(E) vs Area(L) in control (open circle), Area(E) vs Area(L) in DA (filled circle). (E) = early-adaptation; (L) = late-adaptation. F: Bar plots show mean Area and error bars represent S.E. Significant differences (based on paired t tests) are marked by asterisks (*, p<0.02; **, p<0.01).

Fig. 6 (A–D) illustrate the receptive field of a dimming detector in early- and late-adaptation under control and DA conditions respectively. For this neuron, the receptive field in early-adaptation was larger than that in late-adaptation in control; with DA application, despite the shape change, the RF area was increased in both periods as compared to control. For the 17 neurons, the adaptation-dependent reduction of receptive field size was found in 13 neurons in control (Fig. 6E, open circles), with the mean Area(E) and Area(L) significantly different (Area(E) = 17.38±1.50 (a.u.), Area(L) = 13.46±0.97 (a.u.), p<0.01, paired t-test) (Fig. 6F). During DA application, there was no obvious reduction of Area in late-adaptation (Fig. 6E, filled circles). The mean size of receptive field showed no significant difference (Area(E) = 19.76±1.25 (a.u), Area(L) = 20.15±1.42 (a.u.), Fig. 6F). Meanwhile, during DA application, the mean size of receptive field increased significantly both in early- and late-adaptation, as compared with control (Fig. 6F). These results are well compatible with the results of synchronization strength (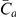).

D1 and D2 dopamine receptors were found to be effective in modulating the coupling among RGCs [38]. To examine the role of D1/D2 receptors in DA-induced enhancement in synchronized activity, we applied D1 and D2 receptor antagonists in contrast adaptation experiments.

**Figure 7 pone-0034336-g007:**
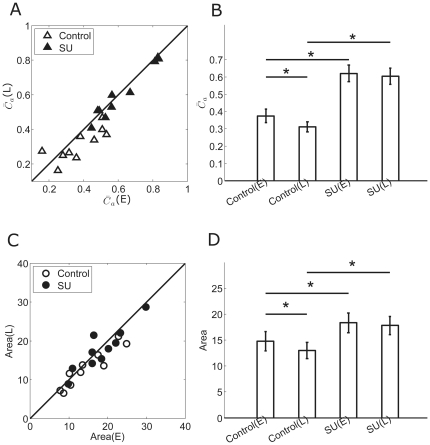
The synchronization index and RF changes measured during sulpiride application. A: The comparison of synchronization index in early- and late-adaptation during control (open triangles) and sulpiride application (filled triangles). SU = sulpiride. B: Bar plots showing mean synchronization index for control and sulpiride application respectively (mean ± S.E., paired t-test, *, p<0.05). (E) = early-adaptation; (L) = late-adaptation. C: The comparison of RF measured in early- and late-adaptation during control (open circles) and sulpiride application (filled circles). D: Bar plots showing mean RF area for control and sulpiride respectively (mean ± S.E., paired t-test, *, p<0.05).

Fig. 7 shows the results of D2 receptor antagonist sulpiride (SU, 10 µM) application. Data were collected from 10 RGCs in 4 retinas. During control, the synchronization index was significantly decreased along adaptation (Fig. 7A, B, 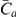
(E) = 0.38±0.04, 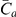
(L) = 0.31±0.03, p<0.05, paired t-test). Significant decrease was also observed in RF area (Fig. 7C, D, Area(E) = 14.78±1.89 (a.u.), Area(L) = 12.99±1.55 (a.u.), p<0.05, paired t-test). Application of sulpiride produced a significant increase in the synchronization index in both early- and late-adaptation (Fig. 7A, B, 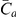
(E) = 0.62±0.05, 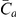
(L) = 0.60±0.05), and for RF area, significant increase was also observed (Fig. 7C, D, Area(E) = 18.31±1.87 (a.u.), Area(L) = 17.81±1.75 (a.u.)). In addition, sulpiride eliminated the decreases of 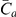 and RF size between the early- and late- adaptation periods (Fig. 7B, D).

In this set of experiment, the application of sulpiride inhibited D2 receptors whilst allowed D1 receptors being activated by endogenously released DA. Thus, it suggests that the enhancement on gap junction connection might be dependent on D1 receptor-dependent cascade.

**Figure 8 pone-0034336-g008:**
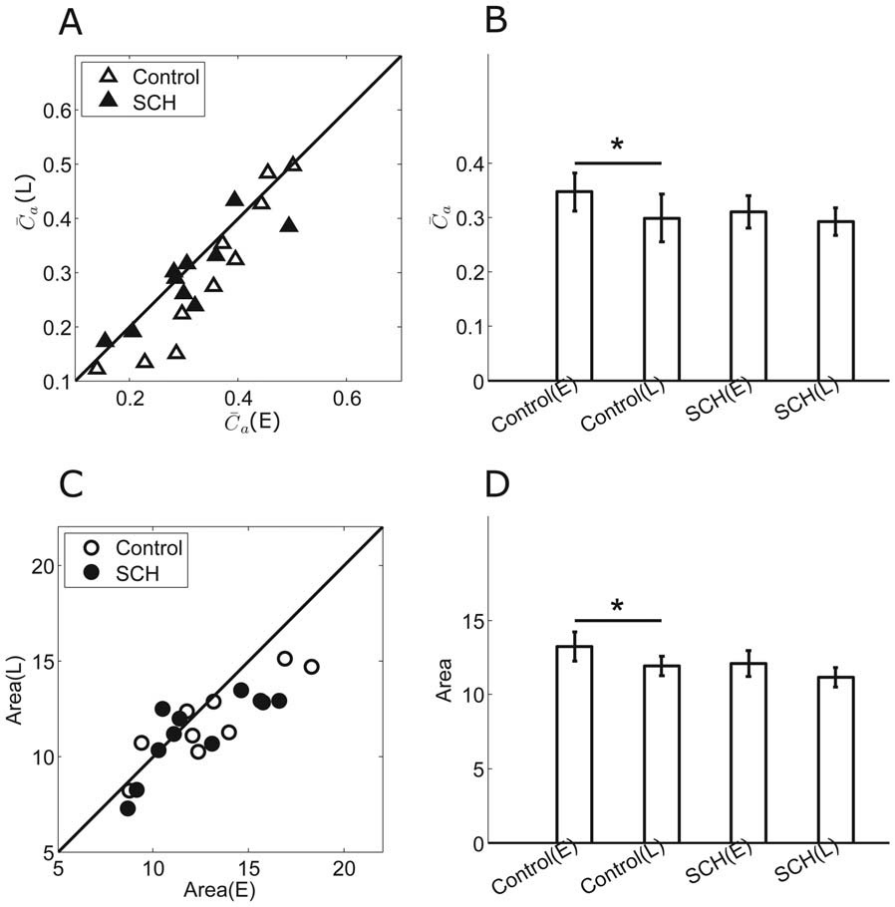
The synchronization index and RF changes measured during SCH-23390 application. A: The comparison of synchronization index in early- and late-adaptation during control (open triangles) and SCH application (filled triangles). SCH = SCH-23390. B: Bar plots showing mean synchronization index for control and SCH-23390 respectively (mean ± S.E., paired t-test, *, p<0.05). (E) = early-adaptation; (L) = late-adaptation. C: The comparison of RF in early- and late-adaptation for control (open circles), SCH-23390 (filled circles). D: Bar plots showing mean RF area for control and SCH-23390 respectively (mean ± S.E., paired t-test, *, p<0.05).

To further clarify the role of DA receptors in synchronization modulation, D1 receptor antagonist SCH-23390 (SCH, 10 µM) was applied. Fig. 8 shows the results of 10 neurons from 3 retinas. During control, the adaptation-dependent decrease in both the synchronization index and RF area was significant (Fig. 8A–D, 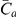
(E) = 0.35±0.04, 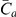
(L) = 0.30±0.04; Area(E) = 13.23±0.97 (a.u.), Area(L) = 11.93±0.66 (a.u.), p<0.05, paired t-test). With SCH-23390 application, the synchronization index and RF showed insignificant decrease in both early- and late-adaptation, as compared with control (Fig. 8B, D, 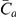
(E) = 0.31±0.03, 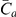
(L) = 0.29±0.03; Area(E) = 12.09±0.87 (a.u.), Area(L) = 11.14±0.65 (a.u.)), and the significant adaptation-dependent decrease between the two adaptation periods was attenuated when D1 receptors were blocked and only D2 receptors were activated by endogenous DA (Fig. 8B, D). This indicates that activation of D2 receptors by endogenous DA release caused weak decrease in gap junction connection, and could not trigger a significant adaptation-dependent variation in synchronization and RF area.

**Figure 9:**
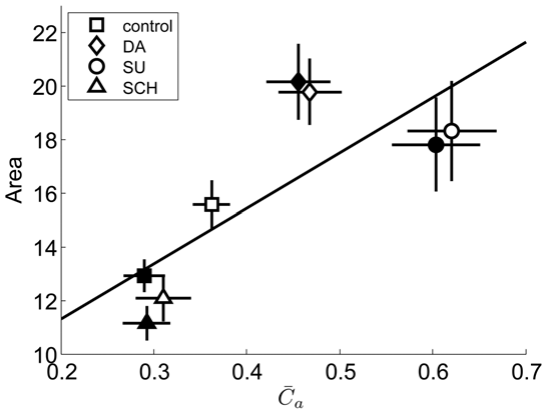
The results of pharmacological experiments showing the relationship between RF area and 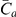. Symbols denote the mean values (open symbols: early-adaptation; filled symbols: late-adaptation) during control (squares), and application with DA (diamonds), SU (circles) and SCH (triangles). Error bars denote S.E.. Correlation coefficient R = 0.78.

These results, together with the DA/sulpiride experiments, confirm that DA-induced increase in synchronization was due to activation of D1 receptors, and RF area was linearly correlated with the strength of the synchronous activity (Fig. 9, correlation coefficient R = 0.78).

## Discussion

Receptive field size and synchronous activities in different adaptation periods were investigated in the present study. It was found that both receptive field size and synchronization strength exhibited adaptation-dependent decrease (Figs. 3 and 4), but both of these decreases were eliminated during DA-application, which reveals that strong gap junctional coupling is related to the receptive field extension in retinal ganglion cells (Figs. 5 and 6), and dopamine-induced modulation is a potential mechanism for gap junction strength changes during population adaptation process [18], [33], [39], [40], [41]. The dopamine-related adaptation phenomena have been extensively described in “illumination adaptation” [33], and the results found in “contrast adaptation” here might be a useful supplement to the understanding of dopaminergic pathway function in adaptation.

### Contrast adaptation of the population activity

For RGCs, adaptation to contrast stimulus refers to the phenomenon that the neuron's response is gradually decreased in exposure to sustained contrast stimulus. In our experiment, the flickering checkerboard was considered as constant contrast stimulation [29], [42]. In this way, the mean intensity was kept constant over time, while the stimulus contrast was set as 100%. In the present study, we were looking into the adaptation process during the retinal ganglion cells' response to 250-s constant-contrast stimulation. It was observed that it took about 50 seconds for the cells' firing rate to decrease and reach a steady state (see Figure 3 (A), Figure 5 (A, B)), which is longer than the adaptation process observed by Baccus and Meister (2002) using a rapidly flickering uniform field whose light intensity changed randomly every 30 ms with a Gaussian distribution. “Early/late adaptation” was used in our work to describe the two different stages during the adaptation process to measure the retinal ganglion cell's response property changes related to contrast adaptation, and the division of the two parts was related to the dynamics of the adaptation process. Although the “early-adaptation” defined here covers the “early- and late-adaptation” defined by Baccus and Meister (2002), but adaptation is stimulation protocol dependent, different stimulation causes different adaptation dynamics, which may lead to the changes of “early” and “late” adaptation duration. The intrinsic property of “early/late-adaptation” is characterized by obvious decline and steady-state in firing rate respectively (Figure 3(A)), which is compatible with the concept adopted by Baccus and Meister (2002).

In our experiment, the transition from darkness to checkerboard flickering also causes mean light elevation in addition to contrast stimulation. To make things clear, we conducted some additional experiments with modified stimulation that a 10-s full-field light (light intensity = (*I*_max_+*I*_min_)/2) was applied before the “contrast adaptation” during the retinal response to sustained checkerboard flickering. The light intensity of the full-field light was set to the same value as the mean light intensity of the checkerboard flickering, which led to “light adaptation” of the RGCs before the “contrast adaptation” during the retinal response to checkerboard. It was found that, in spite of the elevated mean light intensity, the properties of “contrast adaptation” we have been focused on still hold (data not shown).

Neuronal population activity is of significant importance in retinal information encoding and transmission [43], [44]. In our present study, we were particularly interested in the adaptation of population activity, and noticed that synchronous activity among dimming detectors was reduced during the adaptation process (Fig. 4), which provides the evidence that the population activity also exhibits adaptation-dependent variation and dopaminergic pathway participates in the modulation.

### Gap junctional coupling contributes to the extension of receptive field

In the vertebrate retina, neurons of the same type are often extensively coupled through gap junction as demonstrated by the intercellular movement of various tracers [11], [35], [45], [46], [47], which suggests that the larger size of receptive field which exceeds the corresponding dendritic field is probably due to the extensive gap junctional coupling within the network [10], [12], [13]. Besides, action potentials can be evoked in a ganglion cell by its coupled neighbors [48], which makes synchronized activity an additional candidate for presynaptic excitatory input and a reasonable component for “receptive field” [14].

Such relationship between the strength of synchronization and the extension of RF is also confirmed by our experimental observations. Our data show that RF size of the dimming detectors is related to the variation in their synchronous activity (Figs. 5–9). For a neuron, the degree of synchronized activity with its neighbors reflects the strength of gap junctional coupling among them, and the ion current as lateral input via gap junction provides alternative excitatory drive, therefore regulates the formation of the RF of a dimming detector. However, it should be noted that the degree of dependence for RF on synchronous activity is different among retinas, which primarily accounts for the variability in data as showed in Fig. 4(D). The “dimming cell subtypes” may cause the data variability as well [31], [49].

Synchronous activity contributes to the extension of the single neuron's receptive field, consequently helps to remain a high sensibility of the retinal ganglion cells in response to stimulation. On the other hand, the extension of receptive field induces blurring in vision [18]. This is to say that accompanied with the strong synchronization, the extended receptive field is supposed to keep the retinal neurons sensitive enough, which allows for an immediate response to the visual stimulation, but may not get a clear detailed picture of the scene [26]. Along with the adaptation to sustained stimulation, the adaptation-dependent reduction of synchronization and the consequent shrinkage of receptive field size might attenuate the blurring and bring in functional benefits, since relatively weak lateral signal propagation might sharpen the visual acuity and be helpful for sensing finer structure of visual scene and processing more detailed spatial information. In fact, it has been reported that, in the macaque primary visual cortex (V1) neurons, the adaptation-dependent reduction in the strength of correlation caused an improvement in the efficiency of population coding [50], suggesting that decorrelation of neuronal activities may lead to improved information sampling of the stimulus in individual pathways.

### Mechanism of dopamine-induced changes

The light-activated neuromodulator dopamine (DA), which is released by interplexiform cells in the inner retina [40], activates a number of intracellular pathways involving cAMP-dependent protein kinase. This results in some modification of the gap junction connexins, and changes the gap junctional permeability to ionic currents [18].

Our results showed that, with exogenous DA-application, synchronous activities among dimming detectors were significantly enhanced, and the synchronization strength was kept unaltered during adaptation, suggesting that exogenous DA-application increased the conductance of gap junction between dimming detectors, and eliminated the adaptation-dependent reduction in synchronization of population activities.

D1 and D2 dopamine receptors exert opposite regulatory effects on intracellular phosphorylation and gap junction permeability [18]. In our results, application of D2 receptor antagonist resulted in an increase in synchronization which was similar to that observed in DA application. Application of D1 receptor antagonist could not induce a significant change in synchronization. We speculate that DA-induced increase in gap junctional conductance was due to D1 receptor activation. On the other hand, D2 receptor, activation of which counteracted the increase induced by activation of D1 receptor, was inhibited during DA application. It was also reported that increased DA concentration was likely to desensitize D2 receptor [38], [51], which could result in increase in gap junctional conductance via D1 receptor activation [38]. Overall, based on our pharmacological results and other studies, including Hu, Bloomfield (2010) and Mills (2007), we infer the mechanism as follows: activation of D1 receptors elevates intracellular cAMP which phosphorylates connexins via PKA pathway, and results in an increase in the channel conductance and ganglion-ganglion coupling. Activation of D2 receptors has an opposite action on cAMP production, which induces dephosphorylation of connexins and decreases gap junctional coupling. The high-concentration-DA-induced synchronization enhancement could be attributed to both D1 receptor activation and D2 receptor inhibition.

DA analogs can also affect coupling of other types of retinal neurons. The hemichannels (connexon) in amacrine cell are connected with the hemichannels in RGCs [9], and the ganglion-amacrine coupling enables two RGCs to receive a common input from an amacrine cell, which produces correlated activity between the two RGCs' responses and is characterized by the mediate-width (about 50 ms) central peak of the cross-correlogram function for the two RGCs' spiking sequences. The strength of mediate-width correlation is therefore related to the ganglion-amacrine coupling, and we calculated the mediate-width correlation between RGCs' responses in pharmacological experiments as a measure of the hemichannel's open state of amacrine cell (data not shown). The results suggested that the conductance of hemichannel in RGC was increased by DA&SU and slightly decreased by SCH, and the conductance of hemichannel in amacrine cell was probably modulated to the opposite direction. The combination of the two opposite modulated hemichannels produced weakened conductance of the complete channel, and consequentially the decreased correlated activity. Thus the RF extension by DA application here was not due to strengthened excitatory input from amacrine cells. In addition to amacrine cells coupled with ganglion cells, other upstream sites such as bipolar cells also have a potential for providing common input to ganglion cells, which contribute to the wide correlation with distributed time lags (correlated firings) [52]. These cells also express dopamine receptors. However, the strength of such correlated firings, as the outcome of the overall upstream common input, was decreased by DA, SU and SCH (data not shown), showing that correlated firings caused by common input are not positively related to the RF modulation.

Horizontal cells in carp [53], white bass [54], mudpuppy [55], rabbit [56], mouse [57] and macaque [58] retinas were found to be uncoupled by endogenous and exogenous DA. It is well accepted that horizontal cells mediate lateral inhibition in the outer retina, giving rise to the antagonist surround of the RGC's receptive field, and the decoupled horizontal cells network by DA may weaken the antagonist surround and produce less inhibitory effects on the receptive field center, then lead to receptive field center enlargement. However, the receptive field of the dimming detectors had no obvious antagonist surround [31], and the presumed weakened antagonism caused by decoupled horizontal cells may not account for the extended RF by DA here. Accordingly, Dedek et al (2008) compared the effects of horizontal cell coupling on the RGC's receptive field between wild-type and connexin57-knockout mouse, and showed that the coupling and uncoupling of horizontal cells exhibit no different effects on the architecture of the RGC's receptive field [59].

Ribelayga, C. (2008) showed that, in goldfish and mouse retinas, the decoupling of rod-cone gap junctional network in the day time was caused by the high extracellular levels of DA induced by photopic light [35]. Therefore, we speculate that the RF expansion with DA application here is unlikely related to the rod-cone coupling.

In addition to DA-dependent gap junctional modulation, DA also has multiple trophic roles in retinal function related to circadian rhythmicity, cell survival and eye growth [33]. Although we can not rule out the potential effects of other DA-related pathway that is involved in the modulation of synchronous activity, the effect on inter-neuronal gap junction is the most effective way for DA to regulate the synchronous activity between RGCs.

Exposure to light stimulation greatly increased dopamine production in retinas [34], [36], [60]. Based on DA-induced RF expansion observed in our experiment, the following mechanism can be inferred as responsible for the adaptation-dependent changes in synchronization: in early-adaptation, light-induced increase in dopamine release activated D1 receptors and desensitized D2 receptors. Thus, the gap junction conductance between ganglion cells was enhanced to a relatively high level, resulting in stronger synchronization. In late-adaptation, as DA release was declined (for reference, also see [60]), the decrease in D1 receptors activation and probable recovery of D2 receptors from desensitized status in low dopamine concentration both induced the decrease of gap junctional conductance.

In summary, RF and synchronization investigated with DA analogs in the present study are quite consistent in indicating that DA would be an important neuromodulator participating adaptation-related modulation in population activity of retinal ganglion cells, which plays an important role in balancing neurons' capabilities of immediate response and encoding details of the visual scene.
